# Understanding the experiences of Black Nova Scotians with community pharmacists

**DOI:** 10.1177/17151635231202754

**Published:** 2023-10-12

**Authors:** Afomia Gebre, Susan Bowles, Laura V. Minard, Barbara Hamilton-Hinch, Natalie Borden

**Affiliations:** Pharmacy Department, Nova Scotia Health-Central Zone, Halifax; Pharmacy Department, Nova Scotia Health-Central Zone, Halifax; College of Pharmacy, Faculty of Health, Dalhousie University; Department of Medicine, Faculty of Medicine, Dalhousie University, Halifax, Nova Scotia; Pharmacy Department, Nova Scotia Health-Central Zone, Halifax; School of Health and Human Performance, Faculty of Health, Dalhousie University; College of Pharmacy, Faculty of Health, Dalhousie University

## Abstract

**Background:**

A history of medical abuse and social inequality confounded by persistent racial discrimination in health care has triggered mistrust between Black patients and health care providers. Although the consequences of systemic racism on health outcomes are well understood, little is known about how they manifest in pharmacy practice. The objective of this study was to explore the experiences of Black Nova Scotians with community pharmacists.

**Methods:**

This was a qualitative study that used focus groups and one-on-one interviews. Black Nova Scotians 18 years of age and older who have had interactions with community pharmacists were invited to participate. Focus groups and interviews were audio-recorded, transcribed and analyzed thematically.

**Results:**

Two focus groups (*n* = 10) and 6 one-on-one interviews were held between May and June 2021. Three major themes were identified: 1) difficulties navigating a pharmacy as a Black person, 2) lack of inclusivity and cultural competence in the pharmacy and 3) transactional relationships with pharmacists.

**Discussion:**

Most participants felt their race negatively affected the quality of care they received from the pharmacist and that pharmacists were not culturally competent. Most participants did not consider pharmacists to be part of their health care team and described feeling unsafe or uncomfortable in the pharmacy.

**Conclusions:**

Pharmacists have an important role in closing the health equity gap. This research highlights the need for pharmacy education to include cultural competence and will be used to guide strategies to improve access to culturally safe pharmacy services for Black Nova Scotians.

## Introduction

Social, economic, environmental and structural disparities contribute to health inequities between and within communities.^1,2^ Those who are socially disadvantaged have less access to health services, suffer from more illnesses and die at a younger age.^3^ Due to unjust social structures and institutionalized racism, Black patients experience enduring and pervasive health disparities. Cultural distrust between Black patients and health care providers has been triggered by a history of medical mistreatment and social inequity.^4^ Positive health care outcomes are dependent upon interpersonal trust between patients and health care providers, as well as social trust in health care organizations.^5^ Related to interpersonal trust is the acceptance of recommended treatment, satisfaction with care and self-reported health improvement.^6^ Given the expanding scope of pharmacy practice, interpersonal trust between patients and pharmacists has become particularly important.

**Knowledge into Practice**

- As the role of pharmacists continues to expand and they become even more integrated in the patient care process, it will be extremely important for pharmacists to have the knowledge to provide culturally safe services to Black patients.
- Black participants felt that their skin colour negatively affected the quality of care they received from pharmacist and pharmacy team members.
- Participants did not feel that pharmacists have the knowledge to deliver culturally safe care.
- Many Black patients do not consider their pharmacist to be part of their health care team and view the relationship with their pharmacist as strictly transactional.
- Further research is pivotal to identify the way in which systemic racism manifests in pharmacy practice

Pharmacists have the opportunity to play a major role in addressing unmet patient care needs to reduce some pressure on the health care system as it faces new challenges.^7,8^ In Nova Scotia, the importance of pharmacists in the health care system has increased due to the burden of COVID-19, lengthy emergency room wait times and a shortage of primary care providers.^9,10^ To alleviate some of these stressors, pharmacist-led health care clinics have been established in Nova Scotia. These clinics offer services such as managing chronic diseases (e.g., diabetes and asthma), as well as diagnosing and treating some acute conditions (e.g., urinary tract infections and strep throat).^11^

As the role of pharmacists expands, the delivery of culturally safe care for Black patients using these services needs to be a priority. Although systemic differences in health care delivery and their effects on health outcomes are well recognized, little is known about how these manifest in pharmacy practice. Given the scarcity of research in this area, we aimed to explore the experiences of Black Nova Scotians with community pharmacists.

## Methods

### Study design

This was a qualitative study that used focus groups and one-on-one interviews. This dual approach was used due to availability of participants during a substantial wave of COVID-19 in our jurisdiction. Interviews and focus group sessions took place virtually on Zoom for Healthcare, which has additional privacy measures to protect the identities of participants. Sessions were audio-recorded, transcribed and then analyzed thematically. This study was approved by the Nova Scotia Health Research Ethics Board (REB 32435).

**Mise En Pratique Des Connaissances**

- À mesure que le rôle des pharmaciens continue de s’élargir et qu’ils sont de plus en plus intégrés dans les processus de soins aux patients, il sera extrêmement important pour les pharmaciens d’avoir les connaissances nécessaires pour fournir des services culturellement sûrs aux patients de race noire.
- Les participants de race noire estimaient que la couleur de leur peau avait une incidence négative sur la qualité des soins qu’ils recevaient des pharmaciens et des membres de l’équipe pharmaceutique.
- Les participants ne pensaient pas que les pharmaciens avaient les connaissances nécessaires pour fournir des soins culturellement sûrs.
- De nombreux patients de race noire ne considèrent pas leur pharmacien comme faisant partie de l’équipe soignante et estiment la relation avec leur pharmacien comme étant strictement transactionnelle.
- Il est essentiel de poursuivre les recherches pour déterminer la manière dont le racisme systémique se manifeste dans la pratique de la pharmacie.

### Participants

Black Nova Scotians at least 18 years of age who have had interactions with community pharmacists were invited to participate in this research. Flyers advertising the study were posted on social media platforms such as Facebook, Instagram and Twitter. Local and provincial organizations such as Promoting Leadership in Health for African Nova Scotians (PLANS), Delmore Buddy Daye Learning Institute (DBDLI), Imhotep’s Legacy Academy (ILA) and the Pharmacy Association of Nova Scotia (PANS) were contacted to distribute the study flyer. In total, 16 volunteers (*n* = 16) participated in this study.

### Semi-structured interviews and focus group moderation

Interviews and focus group sessions were conducted by the principal investigator and lasted a maximum of 2 hours. Interviews consisted of semi-structured, open-ended questions (Appendix 1). Prior to the focus group sessions and one-on-one interviews, informed consent, demographic data and pledge of confidentiality forms were completed.

### Data collection and analysis

Interviews and focus group sessions were audio-recorded and transcribed by the principal investigator. Field notes were completed after each focus group and interview to highlight key words or phrases expressed by participants. NVIVO 12 software was used to facilitate data analysis.^12^ Data were analyzed thematically using a constant comparative method to compare responses of participants in each focus group and individual interviews. This involved identifying codes, combining codes into categories and then identifying themes.^13^ Thematic analysis was conducted by the principal investigator and reviewed by the research team.

### Rigour and trustworthiness

Measures were taken to increase the trustworthiness of this research. A reflective journal was used during data collection and analysis to record thoughts and codes, as well as identify any assumptions and biases with potential to affect the data analysis process.^14^ Peer debriefing sessions with research team members were also held to further explore the data, provide new insight and challenge any interpretations made by the principal investigator.^15,16^ Anonymous quotes from participants were used to show the authenticity of the findings. Last, an audit trail was kept to document the development of the completed analysis of the data.^17^

## Results

Two focus groups, each with 5 participants and 6 one-on-one interviews (*n* = 16), were conducted between May and June 2021. The majority of participants were female and between the ages of 18 and 35, and most had resided in Nova Scotia since birth. All participants had completed formal education at the college or university level. A comparison of the number of new codes that emerged from each session is shown in Table 1. Three major themes, 9 categories and 54 codes were identified. Code overlap between focus groups and interviews is presented in Table 2. The 3 major themes were as follows: difficulties navigating a pharmacy as a Black person, lack of inclusivity and cultural competence in the pharmacy and transactional relationships with pharmacists (Table 3). A list of illustrative quotes organized by themes and categories is given in Table 4.

**Table 1:** Comparison of codes between focus group sessions and one-on-one interviews

	Focus group 1	Focus group 2	Interview 1	Interview 2	Interview 3	Interview 4	Interview 5	Interview 6	Total
Number of participants	5	5	1	1	1	1	1	1	16
New codes	35	10	4	3	0	0	0	2	54
Total number (%) of codes (54)	35 (65)	29 (54)	27 (50)	36 (67)	24 (44)	26 (48)	25 (46)	22 (40)	

**Table 2:** Code overlap between focus group sessions and one-on-one interviews

	Focus group 1 and focus group 2	Focus group 1 and interviews	Focus group 2 and interviews
Code overlap, *n* (%)	20/35 (57)	34/35 (97)	27/29 (93)

**Table 3:** Overview of themes and categories in the study

Theme	Categories
Difficulties navigating pharmacy as a Black person	• Consciousness of Blackness • Microaggressions and stereotyping • If I was white it wouldn’t be this complicated
Lack of inclusivity and cultural competence in the pharmacy	• Lack of representation • Cultural competence education for the pharmacist • Building genuine relationships with Black patients
Transactional relationships	• Lack of relationships with pharmacists • Lack of privacy in the pharmacy • Loyalty

**Table 4:** Illustrative quotes by themes and categories

Theme	Category	Illustrative quote
Difficulties navigating the pharmacy as a Black person	Consciousness of Blackness	“I was just thinking that . . . when I have really good health care, I’m like, ‘Oh my God, that is amazing.’ This is great but I know that is the bare minimum for so many other people. Whereas I’m like, wow, this person, you know, made me feel like a real person—didn’t . . . just treat me like a symptom and all this stuff. We are conditioned to accept what we get because we are getting something and as long as it is not outwardly racist.”
	Microaggressions and stereotyping	“They definitely have a preconceived notion that people of colour don’t have enough or [aren’t] in the situation to pay for it [medication].”
	If I was white it wouldn’t be this complicated	“I do understand if I was white it probably wouldn’t be this complicated. It would just go smoother, so I think it is very based on our skin colour.”
Lack of inclusivity and cultural competence in the pharmacy	Lack of representation	“You get a lot of pharmacists who are white and nothing against them but it adds to that barrier . . . that mindset of medical treatment in general which is like . . . that level of slight hierarchy because I am the client and you’re the pharmacist so there is a power difference already because I know that you have something that I need but also generally . . . even if it is just the white coat syndrome of seeing a medical professional and having that bit of apprehension because you’re like this is not a space I navigate through all the time but when you see . . . diversity, it feels more reflective of yourself or your community and adds a level of inclusion that breaks the barrier . . . of that power dynamic.”
	Cultural competence education for pharmacists	“I think the non-Black pharmacists, if they don’t actually go through this already, could benefit from some . . . antioppression or kind of cultural awareness or even just like basic intro to psychology type training stuff.”
	Building genuine relationships with Black patients	“I was gonna say . . . sincere, like outreach. Not performative. If there was some substance behind reaching out to the Black community. Do they even know this is an issue? I think it’s amazing you’re doing this research but I’m also like you know, these pharmacies have been here for how long? How come this research has never been done? Like you’ve had Black customers. You know?”
Transactional relationships	Lack of relationships with pharmacists	“I think of pharmacists almost like how I think of the person at Tim Hortons. I am going to get my coffee and that’s it. Up there for 2 seconds to grab my coffee and then I’m going. I don’t sit there and ask where they get their coffee beans from or what is this syrup that you put in your . . . you know what I mean? So same thing, like I’m there to get my prescription and then go.” “I don’t like going in somewhere and greeting them and asking how they are and they immediately ask for my name.” “Her perspective should be from ‘do I have an allergy to it’ or if it’s a duplicate or is there a doctor slip up where I am being double prescribed and that should be her perspective for her role in filling my prescription. We all have a role and position to play.”
	Lack of privacy	“I just feel like the design of pharmacies doesn’t provide you with the opportunity to have conversation because there’s no sense of privacy given to you.”
	Loyalty	“I will say most of my positive experiences have been at my pharmacy with my family pharmacist.”

### Difficulties navigating a pharmacy as a Black person

Many of the conversations in the focus groups and one-on-one interviews were centred on the barriers and difficulties of navigating the pharmacy as someone who is visibly Black. Categories under this theme were Consciousness of Blackness, Microaggressions and stereotyping and If I was white, it wouldn’t be this complicated.

#### Consciousness of Blackness

“The moment that I wake up and I interact or talk to someone, I’m always like hypervigilant of the fact that I’m Black. Yep, so it’s like being careful by the way that I present myself and what I say and how I say it and how people are going to perceive me.”

Participants felt that their race negatively affected the quality of care they received from pharmacists. In many areas of their lives, they described looking at themselves through the eyes of a racist society and this was no different in the pharmacy. This heightened awareness added a layer of anxiety, as many of them had fears of being perceived negatively, which could affect the care that they receive.

#### Microaggressions and stereotyping

Microaggressions and stereotypes were identified as contributing to the poor quality of care received by participants from pharmacists. These instances were often described as being subtle and woven into the routine at the pharmacy. Female participants described having to police their emotions within the pharmacy out of fear of being labeled as the stereotypical “angry Black woman.” Other stereotypes described by participants included drug-seeking behaviour or being less educated or poor. In anticipation of discriminatory behaviour from pharmacy team members, participants described changing the way they spoke and behaved. Most felt that if they did not change the way in which they spoke, they would be labelled as uneducated and information could be withheld from them because the pharmacist might assume that they would not understand it.

#### If I was white, it wouldn’t be this complicated

A significant amount of time in each of the interviews and focus group sessions was spent discussing the way in which race complicated the pharmacy experience for participants. They described how even completing a simple task at the pharmacy, such as picking up a prescription, left many of them feeling drained. For participants, there was a sense of mental preparation that needs to happen before going into the pharmacy. Additionally, due to their lack of trust in the health care system, many participants were skeptical of the information provided by health care practitioners.

### Lack of inclusivity and cultural competence in the pharmacy

This theme included the categories Lack of representation, Cultural competence education for pharmacists and building genuine relationships with Black patients.

#### Lack of representation

The lack of Black pharmacists and pharmacy team members was an issue highlighted by all participants. It was discussed that the health care system was not made for Black people to thrive in and how lack of inclusivity and diversity reinforces this idea for many. Only 1 participant in this study had had an interaction with a Black pharmacist before. All participants felt that diversity was crucial for creating safe spaces for Black participants. Many stated that having a Black pharmacist would change the experience for them in many ways. They would feel a more genuine connection with the pharmacist. They also wouldn’t feel like they had to change the way they spoke or presented themselves, also known as code switching, in the pharmacy. Participants also explained that they would feel understood because of shared experiences and trauma. For participants, even having a pharmacist who was of a racially visible minority, not just Black, would make the space feel safer and more accessible. This would help them feel more comfortable asking questions, building relationships and learning more about the role of a pharmacist.

#### Cultural competence education for pharmacists

All participants felt that pharmacists are not equipped with the education to provide culturally safe care for Black people and emphasized the importance of pharmacists and pharmacy learners to complete antiracism and antioppression education. Many highlighted that this education would also help minimize microaggressions and make practitioners aware of their implicit biases and internalized racism. Additionally, many felt it was important for pharmacists, especially those who work in proximity to historical African Nova Scotian communities, to become educated on the history of these communities.

#### Building genuine relationships with Black patients

Participants discussed that Black communities are often skeptical of outreach from health care practitioners due to mistrust between the community and the health care system. They emphasized that any effort to build this relationship must be genuine and not performative. To ensure the interaction is safe, participants highlighted that pharmacists need to make themselves aware of their implicit biases and make strides towards unlearning these beliefs before reaching out to Black people. Participants felt that this would result in genuine efforts to create safe spaces for Black people within the pharmacy, leading to improved relationships with the pharmacist. They also envisioned that this awareness would help create fair opportunities for all people accessing the pharmacy, as many participants believed that instances of being treated poorly stemmed from pharmacists not being aware of their internalized racism.

## Transactional relationships with pharmacists

This theme included the categories Lack of relationships with pharmacists, Lack of privacy in the pharmacy and Loyalty.

### Lack of relationships with pharmacists

The transactional relationship between participants and their pharmacist was a major discussion item. Most participants spoke about not having a relationship with their pharmacist and seeing them as simply a bridge between their doctor and their medications. When asked who their first contact would be if they had questions about their medication, most participants said they would either try to contact their doctor or look the information up on Google. When probed further, some participants explained they would never contact their pharmacist with a question pertaining to their medication and others listed pharmacists as a last line option. When asked if they saw their pharmacist as part of their health care team, most participants said no.

A major contributor to participants seeing the relationship with their pharmacist as strictly transactional was that many considered pharmacists to be cold, too clinical and lacking empathy. Participants described leaving the pharmacy feeling frustrated and dismissed due to the lack of connection between them and their pharmacist. Many participants recognized that pharmacists were busy and that the pharmacy seemed understaffed; however, they felt that if their family doctor was able to make a genuine effort to build a relationship with them, then why couldn’t their pharmacist? When asked about medication counselling, some participants did not understand the importance of these counsels. Most described them as short and rushed.

There was a lack of understanding among participants of the role and scope of practice of a pharmacist. For example, many did not understand the reason for the questions that pharmacists asked, such as the indication for their medications or if they had had the medication before. Due to this lack of understanding, some participants felt that pharmacists were intrusive and overstepping their role.

### Lack of privacy in the pharmacy

One of the main issues for participants was the lack of privacy in the pharmacy. Many described feeling uneasy about receiving medication counselling at the counter. Often, participants discussed that they were unable to pay attention to what they were being told by their pharmacist due to anxiety that someone around them was listening. This was particularly an issue for participants who used pharmacies in small communities. Some participants explained that they had rarely been asked to go into the counselling room. Others explained that the only times they had been offered the counselling room was when it was for a medication or topic the pharmacist thought was sensitive. The lack of privacy coupled with the general sense of uneasiness in the pharmacy made participants feel hesitant to ask questions.

### Loyalty

Most participants spoke about being loyal to 1 pharmacy, mainly because of convenience. However, there were a few participants who were loyal to 1 pharmacy because of long-term relationships they had with particular pharmacists. As a result, participants felt comfortable and safe with these pharmacists. These were the few instances where participants spoke highly of pharmacists and described their relationship as being more than transactional. Some participants referred to these pharmacists as their family pharmacist. Even when participants moved away from these pharmacies, they continued to use them as their regular pharmacy—1 participant would drive for almost an hour to her pharmacy to see her long-term pharmacist. This participant described travelling these distances because she felt respected and attended to by these pharmacists. Participants described not feeling this same safety and comfort anywhere else and therefore would not seek other pharmacists for questions or advice.

## Discussion

Three major themes were identified in this research: 1) Difficulties navigating a pharmacy as a Black person, 2) Lack of inclusivity and cultural competence in the pharmacy and 3) Transactional relationships with pharmacists. These 3 themes refer to the inadequate care provided by pharmacists to Black patients as well as feelings of discomfort and hostility experienced by Black patients accessing community pharmacies.

The theme Difficulty navigating the pharmacy as a Black person is consistent with what is known from the literature for other health care providers such as physicians. This research suggests the following: 1) Black people are significantly more likely to believe their race negatively affects their health care and 2) Black patients are less trusting of their provider compared to white patients, which negatively affects the likeliness of Black people using these services.^18-20^ These findings are similar to what was seen in our study.

Culturally competent care means delivering effective care to patients who have diverse beliefs, values and behaviours.^21,22^ Participants did not feel pharmacists were culturally competent and therefore were hesitant to explore and make use of pharmacists. This is similar to findings from other research with other health care providers that suggests that lack of cultural competence can lead to patient dissatisfaction, lower quality of care and a negative impact on utilization of services and adherence to recommendations from practitioners.^23^ Cultural competence is a crucial component of patient-centred care and is needed for reducing racial disparities in health care.^24^ Many interventions can be used to increase the cultural competency of an organization, such as making it a priority, increasing diversity, involving the community and investigating and reporting disparities that may exist.^25^

One concept that was rarely seen in the literature for other health care practitioners was that some participants described their relationship with the pharmacist as strictly transactional. The literature describes the idea of transactional care within medicine, specifically nursing.^26^ However, this is different from our findings where participants described the role of the pharmacist as being similar to that of a cashier. This may be because of the traditional community pharmacist roles, such as dispensing. Over time, pharmacists have transitioned into a role where they are more integrated into the patient care process and recognized as medication management experts. Despite this expansion in the scope of practice of pharmacists, participants described pharmacists as a bridge between their doctor and their medication. Most participants also described feeling too unsafe or uncomfortable in the pharmacy to explore what else the pharmacist could do for them. Pharmacists often describe themselves as the most accessible health care practitioners; however, if the pharmacy is a place where some do not feel safe enough to explore and learn about all of these options, then who are they accessible to?^7,27^

### Strengths and limitations

This research had several strengths. To our knowledge, this is the first study to explore the attitudes and experiences of Black people with community pharmacists in North America. Participants were interested and engaged, which allowed for the collection of rich, detailed information on a topic that is understudied. This information is relevant to pharmacy practice and provides insights into how practice needs to change to create safe spaces for Black people accessing pharmacy services. The idea that theoretical data saturation was reached is supported by the identification of a total of only 9 codes after all 6 one-on-one interviews (Table 2).

Accessibility was the primary limitation of this study. Advertisements to participate in this research, as well as the interviews and focus group sessions themselves, were conducted virtually due to the COVID-19 pandemic. This means that those who did not have access to a phone or computer and Internet service were not able to participate in this study. Furthermore, with 1 exception, all participants were female. All participants had completed postsecondary education, and the majority were between the ages of 18 and 35. As such, their experiences may differ from others, potentially reducing the generalizability of the results. However, data from studies with other health care professionals examining a wider demographic of Black people show similar results.^28-31^ Additionally, pharmacists work in patient care areas outside of community pharmacy; however, this research only looked at the experiences of Black Nova Scotians with community pharmacists. Last, there may also have been selection bias of individuals who have had bad experiences at community pharmacy.

## Relevance to practice

As health care practitioners, pharmacists have a role in closing the health equity gap. To begin this work, pharmacists must first listen to the needs of those affected by it.^32^ Through listening, work to amend relationships and build trust between Black patients and pharmacy team members can begin, with the goal of improving health outcomes. Participants put forward the following recommendations to improve their experience with pharmacists and to create a safe space within the pharmacy. These recommendations should be used by stakeholders, educators and pharmacists to guide work that needs to be done to improve the experiences of Black Nova Scotians with community pharmacists.

1. Increased representation within the pharmacy by hiring more Black pharmacists, pharmacy assistants and technicians
2. Antiracism and antioppression education for pharmacist, pharmacy staff and learners
3. Community outreach by pharmacists to the Black community
4. Creating safe spaces within the pharmacy for Black customers

## Conclusion

This research describes the lived experiences of Black patients with community pharmacists. It also highlights the fact that in the current pharmacy setting, many pharmacists do not provide culturally safe care to Black patients. Unless effort is made to understand systemic racism, perceived discrimination and the influences of social determinants of health in the context of pharmaceutical care, health disparities will continue to harm Black patients accessing pharmacy services. The task at hand is not to challenge these attitudes but rather to develop ways to build trust, create safe spaces and dismantle the institutionalized and systemic racism that plagues our health care system. ■

## Supplemental Material

sj-pdf-1-cph-10.1177_17151635231202754 – Supplemental material for Understanding the experiences of Black Nova Scotians with community pharmacists Click here for additional data file. Supplemental material, sj-pdf-1-cph-10.1177_17151635231202754 for Understanding the experiences of Black Nova Scotians with community pharmacists by Afomia Gebre, Susan Bowles, Laura V. Minard and Natalie Borden in Canadian Pharmacists Journal / Revue des Pharmaciens du Canada
