# Supplementary material for: Understanding the experiences of Black Nova Scotians with community pharmacists
Source: Can Pharm J (Ott). 2023 Oct 12;156(6):316–23. doi: 10.1177/17151635231202754 (PMC10655799; doi:10.1177/17151635231202754)
Supplement: sj-pdf-1-cph-10.1177_17151635231202754 – Supplemental material for Understanding the experiences of Black Nova Scotians with community pharmacists [file sj-pdf-1-cph-10.1177_17151635231202754.pdf]

APPENDIX 1 Questioning route for focus groups and one-on-one interviews

Welcome

Hi everyone. Thank you so much for taking the time to be part of this study. I want to start off by introducing myself. My name is Afomia Gebre. I am a pharmacy resident, so what that means is that I am a pharmacist who is doing additional training so that I can work in the hospital.

Overview of Topics

Today we are going to be talking about your experiences and attitudes towards community pharmacists. We know that healthcare historically has been one of the most segregated parts of society so the goal of this study is to gain some insight into what the experiences of Black people in Nova Scotia has been like with pharmacists and to help us address gaps in delivery of care that may be present. We are also seeing that pharmacists are able to do more now (prescribe, renew prescriptions and so on) we want to make sure that Black Nova Scotians benefit equally from this expansion in scope. Throughout this discussion, when I say "pharmacist" I am only referring to community pharmacists which are the people who work at drug stores such as Shoppers Drug Mart or Lawton's.

Does anyone have any questions so far?

I wanted to do a technical overview as well — I mean, I expect some technical difficulties just because that's the way these things go…

- I would love it if everyone had their cameras on however please do not feel like you have to. If you are more comfortable or it feels less vulnerable to have your camera off, then you may do that
- So, this should be what you see on our zoom call – you can see at the bottom you have the option to mute/unmute, start, and stop your video as well as leave
- When you click the participant button at the bottom it will give you a list of who is in our focus group today – at the bottom of that list you should see the option to raise your hand, yes or no and so on just to make sure this is true for everyone can you all click "yes" if you see these options
- Awesome okay, in the same area there should be a "more" option and this will give you the option to clear whatever reaction you have so if you clicked yes and now you want to remove that, that is how!
- This is an open discussion so please jump in at any time, or whatever you need but if we notice someone is speaking and you want the floor next, you can use the "raise hand" function so I can see that you had something to say, and I do not skip over you

Does that make sense? Does anyone have any questions so far?

Ground Rules

1. Participation in the focus group is voluntary – if at any time you decide you no longer want to be part of it or even later on if you don't want your data to be used that is completely valid and you can just let me know. However, because we are in a focus group the information that you say up to the point of when you decide to withdrawal may be used because its hard in the data analysis part to separate someone's point that could have led to more fruitful discussion.
2. There are no right or wrong answers, all responses are valid
3. You can choose not to answer any question you are not comfortable with
4. Do not reveal detailed information about your personal health

5.To protect each other's privacy, do not repeat at any aspect of our discussion today with anyone outside of the focus group

6. Speak one at a time
7. Treat everyone's experience and ideas with respect – do not criticize
8. Minimize side conversations
9. Conversations will be recorded to ensure we do not miss anything

Any questions?

Confidentiality
I wanted to also go over confidentiality. The nature of a focus group is such that confidentiality cannot be guaranteed. That being said, I do want to say that we have put many safeguards to protect your privacy such as removing all identifiers when we do the data analysis and publish the results, only I will have access to these recordings, everyone signing the pledge of confidentiality and if we use any direct quotes, we will make sure that it is anonymous.

Reminder
What we discuss today is not an easy topic to discuss. Anytime you talk about the Black experience whether good or bad, it can be emotional. You may recall unpleasant memories that can be uncomfortable. Please know, I will support you throughout and after these focus groups in any way that I can. If there is something you want to add but do not feel comfortable doing it in the focus group, then you can always contact me, and we can speak privately. Do not hesitate if you need to take a break from the discussion or leave. Also, you do not have to have your cameras on during our discussion. Of course, I would love to see all of you but if you are more comfortable turning your camera off, then do not hesitate to do so.

- **Ask questions – if you need clarification**

**Question (opening – 10 minutes):**<br>**Let's jump right in.**	
**Question:** Do you go to a pharmacy close to where you live or somewhere else?	**Prompt**: Do you go to a regular pharmacy or do you use multiple pharmacies?

**<u>Question</u>:** If you feel comfortable answering do you go for a regular prescription or once in a while?	
**<u>Question</u>:** What are your thoughts and feelings (for example, apprehension, comfort) when you walk up to a pharmacy counter to either pick up a prescription, drop one off or to ask a general question?	**<u>Prompt:</u>**<br>- In what ways do those feelings affect the interaction you have with the pharmacy team?<br>- Has there ever been a time where you were very aware of your Blackness to the point that it changed the way you behaved in the interaction with the pharmacist or the team?
**<u>Question</u>:** Some Black people describe a certain level of anxiety when walking through a retail environment, a store of any sort, have you experienced that while moving through a pharmacy? Has that impacted your interaction with a pharmacist?	
When you receive a new prescription, under the laws that pharmacists work under, a pharmacist must offer you a council which is going over the medication with you (in terms of why you are taking it, the side effects and so on).<br><br>**<u>Question</u>:** Have you ever received a council and what has that experience been like?<br>If you haven't, is it offered to you? How do you feel when you have the conversation?	**<u>Prompt:</u>**<br>- If you refuse council, is there a reason why?<br>- How were things explained to you about your new medication?<br>- How well do you feel your questions were answered?<br>- Would you feel comfortable asking for clarification if you didn't answer correctly?<br>- Are you offered a private space?<br>- Has the pharmacist ever called you for follow up or further clarification?<br>- Is it always in person, is it over the phone?
**<u>Question</u>:** Outside of getting a prescription filled, have you gone to the pharmacy for other services or advice? This can be advice about a family member or a child or just anything in general.	**<u>Prompt:</u>**<br>- If you have ever had a question but did not ask for the advice of a pharmacist, why is that?<br>- If someone on the pharmacy team was Black, would this be different if the pharmacist or someone on the team was Black?
**<u>Question</u>:** If you had a question about your medication, who would you contact first?	**<u>Prompt:</u>**<br>-If not a pharmacist: why not?

**<u>Question</u>:** Can you describe any interactions you have had with a Black pharmacist?	**<u>Prompt:</u>**<br>- If you have never had one, how different would your experiences be if the pharmacist you interact with was Black?<br>- Do you think this would change your experience/how would this impact your experience?<br>- How likely would you be to ask for further clarification if you did not understand something if your pharmacist was Black?
Question: Do you see your pharmacist or really any pharmacist that you interact with as part of your health care team?	- Would this be different if they were Black?
**<u>The end</u>: The purpose of this study was to better understand the experiences of Black Nova Scotians and their attitudes towards community pharmacists. Have we missed anything, or did anyone have any last-minute thoughts?**	

Gebre A, et al. Understanding the experiences of Black Nova Scotians with community pharmacists. Can Pharm J (Ott) 2023;156. DOI: 10.1177/17151635231202754.
